# Topical Application of Everolimus Attenuates Lens-Induced Myopia Through mTORC1 Suppression

**DOI:** 10.1167/iovs.66.12.55

**Published:** 2025-09-24

**Authors:** Ruiheng Zhang, Chuyao Yu, Yitong Li, Jiaoyue Dong, Haotian Wu, Yuhang Yang, Xuhan Shi, Wenda Zhou, Hanqing Zhao, Bingyu Cai, Shanshan Wang, Li Dong, Lei Shao, Wei Li, Jost B. Jonas, Wenbin Wei

**Affiliations:** 1 Beijing Tongren Eye Center, Beijing Key Laboratory of Intraocular Tumor Diagnosis and Treatment, Beijing Tongren Hospital, Capital Medical University, Beijing, China; 2 Beijing Ophthalmology & Visual Sciences Key Lab, Beijing Tongren Hospital, Capital Medical University, Beijing, China; 3 Medical Artificial Intelligence Research and Verification Key Laboratory of the Ministry of Industry and Information Technology, Beijing Tongren Hospital, Capital Medical University, Beijing, China; 4 Key Laboratory of Organ Regeneration and Reconstruction, State Key Laboratory of Stem Cell and Reproductive Biology, Institute of Zoology, Chinese Academy of Sciences, Beijing, China; 5 Institute for Stem Cell and Regeneration, Chinese Academy of Sciences, Beijing, China; 6 University of Chinese Academy of Sciences, Beijing, China; 7 Beijing Institute for Stem Cell and Regenerative Medicine, Beijing, China; 8 Rothschild Foundation Hospital, Paris, France; 9 Singapore Eye Research Institute, Singapore National Eye Center, Singapore, Singapore; 10 Privatpraxis Prof Jonas und Dr. Panda-Jonas, Heidelberg, Germany; 11 Beijing Visual Science and Translational Eye Research Institute (BERI), Beijing Tsinghua Changgung Hospital, Tsinghua Medicine, Tsinghua University, Beijing, China; 12 L V Prasad Eye Institute, Hyderabad, Telangana, India

**Keywords:** myopia, everolimus, mTORC1, guinea pigs

## Abstract

**Purpose:**

The mechanistic target of rapamycin complex 1 (mTORC1) signaling has been reported to regulate lens-induced myopia (LIM) in guinea pigs. To address the challenge of delivering lipophilic mTORC1 inhibitors to the posterior eye segment, we developed a novel topical ophthalmic formulation of everolimus, a second-generation rapamycin derivative available only orally, and evaluated its antimyopic efficacy, ocular pharmacokinetics, and safety.

**Methods:**

Vehicle formulations were optimized for delivering everolimus to the RPE–choroid complex. The efficacy of different concentrations of everolimus eye drops was tested in 3-week-old male pigmented guinea pigs that underwent LIM. We examined mTORC1 signaling activation, axial elongation, refractive changes, and fundus morphology. Pharmacokinetics was assessed in guinea pigs and New Zealand white rabbits. Ocular safety was evaluated through slit-lamp and fundus examinations, intraocular pressure measurements, and histologic analysis.

**Results:**

The optimized formulation of everolimus eye drops (0.001%, 0.01%, and 0.1% w/v) significantly attenuated axial elongation by 0.10 ± 0.03 mm (*P* = 0.054), 0.11 ± 0.02 mm (*P* = 0.001), and 0.14 ± 0.03 mm (*P* = 0.001), respectively. The everolimus eye drops also attenuated fundus tessellation, choroidal thinning, and mTORC1 activation. Peak everolimus concentrations in the RPE–choroid complex of guinea pigs ranged from 5.6 to 103 ng/g, with a Tmax of 1 hour. In rabbits, 0.005% to 0.01% everolimus eye drops achieved concentrations in the RPE–choroid complex comparable to the therapeutic levels in guinea pigs. No corneal, lenticular, retinal toxicity, or intraocular pressure alterations were observed.

**Conclusions:**

This novel ophthalmic formulation effectively delivered everolimus to the posterior segment and inhibited myopia progression, supporting its clinical potential for myopia control.

Axial myopia has emerged as the leading cause of irreversible vision loss in East Asia, driven by its progression to sight-threatening complications, including myopic macular degeneration, retinal detachment, open-angle glaucoma, and other complications.^1^^,^^2^ Development and progression of axial myopia and pathologic myopia are characterized by an increase in axial length of the eye. Continuous axial elongation is the most important and potentially modifiable risk factor for the progression of myopic macular degeneration.^3^ With the global prevalence of high myopia projected to surge from 5.2% in 2020 to an estimated 9.8% by 2050, the urgent need for effective interventions targeting axial elongation has become a global ophthalmologic priority.^4^^,^^5^

The pharmacologic interventions have shown clinical advantages as they can attenuate axial myopia alone and can be combined with lifestyle modification and optical interventions to achieve synergistic efficacy.^6^^–^^8^ For now, the only evidence-based pharmacologic intervention for axial myopia is low-concentration atropine.^9^ It significantly slows axial elongation, choroidal thinning, and the development of high myopia.^10^^,^^11^ In our prior mechanistic work, we demonstrated that the mammalian target of rapamycin complex 1 (mTORC1) signaling was involved in the development of a guinea pig model of lens-induced myopia (LIM).^12^^,^^13^ mTORC1 is a key regulator of basic physiological and pathophysiological processes, including cell growth, metabolism, and survival.^14^ In the eyes of guinea pigs with LIM, the phosphorylation level of ribosomal protein S6 kinase β-1 kinase (p70S6K) in the choroid and retinal tissue was significantly increased, indicating an activated mTORC1 signaling. Intravitreal injection of everolimus, a second-generation rapamycin derivative, once a week for 3 weeks, attenuated axial elongation in a dose-dependent manner.^12^ Combining LIM and intravitreal injections of the mTORC1 agonist (MHY1485) further enhanced myopic axial elongation, including choroidal and scleral thinning and development of parapapillary atrophy.^12^^,^^15^ These results showed that mTORC1 is a key signaling pathway in the process of LIM. Furthermore, intravitreal injections of everolimus were not associated with retinal damage, as assessed by histologic staining.^12^ These results showed that everolimus can be a potential pharmacologic intervention for myopia control.

Intravitreal injection of the mTORC1 inhibitor rapamycin has been used to treat noninfectious uveitis in patients.^16^ However, intravitreal injections carry the risk of injection-related infections and iatrogenic injuries. Repeated administration of invasive procedures is particularly challenging in children and adolescents. A recent study developed 0.23% nanoparticle-based rapamycin eye drops, which achieved significant drug concentrations in the retina and choroid.^17^^,^^18^ Based on our previous study, RPE cells were the primary site of mTORC1 activation that regulates lens-induced axial elongation.^12^ Thus, the present study developed a novel cyclodextrin-enabled ophthalmic formulation to deliver everolimus to the RPE–choroid complex. We investigated whether these everolimus eye drops could achieve effective concentrations in the RPE–choroid complex and exert inhibitory effects on the development and progression of LIM.

## Materials and Methods

The study included New Zealand white rabbits weighing between 3 and 3.5 kg and male pigmented 3-week-old guinea pigs weighing between 150 and 180 g. The guinea pig model of LIM was selected to evaluate the efficacy and safety assessments. New Zealand white rabbits were further included for ocular pharmacokinetics and anterior segment safety assessments, as their ocular dimensions approximate two-thirds of the human eye and are widely used for the pharmacokinetics of topical ophthalmic formulations. All animals were reared in cycles of 12-hour light (400–500 lux) and 12-hour dark (∼0 lux) with room temperature maintained at 25°C, and they had free access to food and water. Guinea pigs were group-housed (two to three per cage), and rabbits were housed individually. The study design, including the treatment and care of the animals, was approved and supervised by the Ethics Committee of Beijing Tongren Hospital (TREC2023-KY076). All research protocols and procedures followed the laboratory animal welfare guideline of the Chinese Association for Laboratory Animal Science and the ARVO statement for the use of animals in ophthalmic and vision research.

The study included four experimental protocols:

1. To optimize vehicle formulation for posterior eye delivery, the right eyes of guinea pigs (*n* = 5) were subjected to different vehicle formulations that contained 0.001% everolimus once a day for 7 consecutive days. Guinea pigs have a relatively low body weight, and bilateral administration could increase systemic exposure, potentially influencing drug distribution in ocular tissues and confounding local pharmacokinetic measurements. Thus, the left eye of the guinea pigs remained untreated to minimize the risk of systemic absorption affecting ocular pharmacokinetics.
2. To assess the effect of everolimus eye drops on LIM, guinea pigs were subjected to binocular LIM and received once-daily everolimus eye drops in their right eyes for 3 consecutive weeks, at concentrations of 0.0001% (*n* = 8), 0.001% (*n* = 8), 0.01% (*n* = 8), and 0.1% (*n* = 8). The left eyes of guinea pigs were treated with vehicle solution without everolimus, serving as the self-control.
3. To evaluate the safety of everolimus eye drops, both eyes of guinea pigs without LIM were subjected to eye drops containing everolimus in concentrations of 0.0001% (*n* = 5), 0.001% (*n* = 5), 0.01% (*n* = 5), and 0.1% (*n* = 5). The eye drops were applied once daily for 3 consecutive weeks. Since 0.0001% everolimus eye drops failed to suppress myopia progression in the guinea pig model of LIM, it was excluded from subsequent safety evaluations conducted in New Zealand white rabbits. Thus, both eyes of rabbits (*n* = 3) were subjected to eye drops containing everolimus in concentrations of 0.001%, 0.01%, and 0.1% without LIM. Ocular tolerability and safety were evaluated at the end of the study.
4. To examine the single-dose and repeated-dose pharmacokinetics of topically applied everolimus, the everolimus concentration was examined in the ocular tissues of New Zealand White rabbits (*n* = 3 for 0.5, 1, 2, 4, and 24 hours after eye drops). The single-dose pharmacokinetic analysis examined the everolimus concentration in eight tissue types: whole blood, cornea, aqueous humor, crystalline lens, vitreous humor, retina, RPE–choroid complex, and sclera. The repeated-dose pharmacokinetics only examined the everolimus concentration in the RPE–choroid complex.

### Eye Drop Formulation and Delivery

Everolimus, γ-cyclodextrins, and hydroxypropyl β-cyclodextrins were purchased from GlpBio Technology (Montclair, CA, USA). Methylcellulose (viscosity: 4000 cP), polysorbate 80, Sorensen's modified phosphate buffer, benzalkonium bromide, and EDTA were purchased from Sigma-Aldrich (St. Louis, MO, USA). To select the highest efficacy formulation for delivering everolimus to the ocular segment, a comparative experiment was first conducted on guinea pigs. Different concentrations of hydroxypropyl β-cyclodextrins (0%, 0.5%, 2%, 5%, and 10%), γ-cyclodextrins (5%), polysorbate 80 (0%, 0.5%, 2%, 5%, and 10%), methyl cellulose (0%, 0.25%, and 0.5%), and 0.001% (w/v) everolimus were used to compose several comparative formulations (Table). These ophthalmic formulations were adjusted to a pH of 7.4 and an osmolarity of 290 mOsm. Five guinea pigs were randomly allocated to each formulation and received 25 µL of the formulation as eye drops at about 2:00 PM every day for 7 continuous days. Based on the achieved concentrations, the final vehicle solution was composed of 5% hydroxypropyl β-cyclodextrins, 0.5% methylcellulose, 2% polysorbate 80, 0.05 mg/mL benzalkonium bromide, and 0.1 mg/mL EDTA. The eye drops were stored at 4°C in a refrigerator.

**Table.** Single-Dose Pharmacokinetics of Everolimus Eye Drops in Guinea Pigs and Rabbits

Species/Concentration	Cmax (ng/g)	Tmax (h)	AUC0-24 (ng⋅h/g)
Guinea pigs			
0.001%	5.6 ± 0.5	1	47.6 ± 9.5
0.01%	13.9 ± 7.8	1	194 ± 39
0.1%	103 ± 40	1	1036 ± 228
Rabbits			
0.005%	9.8 ± 2.5	1	166 ± 28
0.01%	12.9 ± 2.4	1	207 ± 49

The everolimus concentration was examined (*n* = 3 for 0.5, 1, 2, 4, and 24 hours after eye drops).

For administering the eye drops, the upper eyelid was gently pulled away from the eye globe, and the formulations were applied into the conjunctival sac of the guinea pigs and rabbits (25 µL per eye). After application, the guinea pigs were gently held by hand, and the rabbits were held by rigid plastic restrainers for 2 minutes. All eye drops were given at a frequency of once per day.

### Lens-Induced Myopia of the Guinea Pig

To experimentally induce axial myopia in experiment protocol 2, we used the negative lens to induce myopia in guinea pigs, as described in detail previously.^12^ We used lenses (polymethyl methacrylate; diameter: 14.9 mm) with a refractive power of −30 diopters and taped them onto the orbital rim of both eyes of the guinea pigs. The animals could freely open their eyes and blink while wearing the lenses. The refractive power of the lenses and their centration were measured and verified before application. All guinea pigs were examined daily to ensure that the lenses were clean and in place; otherwise, the lenses were detached and replaced. All lenses were removed weekly to perform biometric examinations of the eyes. At the end of the experiment, guinea pigs underwent fundus photography, optical coherence tomography (OCT) imaging, axial length, and refraction measurements, followed by humane euthanasia. Ocular tissues were then harvested for mTORC1 pathway activation analysis.

### Measurement of Axial Length, Refractive Error, Choroidal Thickness, Fundus Photography, and Intraocular Pressure

Axial length and refractive error were measured at baseline and the end of each week throughout the 3-week experimental period. Choroidal thickness, fundus photography, and IOP were assessed only at the end of the 3-week experiment.

All guinea pigs underwent OCT imaging of the posterior eye segment without anesthesia (Swept Source-OCT, VG200G; Intalight Ltd., Guangdong, China). During the OCT scanning, an operator gently held the guinea pigs to achieve a steady position, and an 18-line radial scan centered on the optic disc center was taken. The horizontal and vertical scan images were exported. For each guinea pig, the choroidal thickness was measured at eight positions: the horizontal (3-o'clock and 9-o'clock positions) and vertical meridians (12-o'clock and 6-o'clock positions) at distances of one and three horizontal disc diameters from the optic disc center. At each position, the measurement line was perpendicular to the RPE layer.^15^ Fundus photography was performed using a fundus camera with a wide-field mode (ZEISS CLARUS 500; Carl Zeiss Meditec AG, Jena, Germany).

Under topical anesthesia (0.5% proxymetacaine hydrochloride; Alcon, Tokyo, Japan), we measured the axial length by ocular ultrasonography (A-scan mode scan; oscillator frequency: 11 MHz; Quantel Co., Les Ulis, France). The ultrasound velocities used were 1557.5 m/s for the cornea and aqueous humor, 1723.3 m/s for the lens, and 1540 m/s for the vitreous cavity.^19^^,^^20^ Five measurements were performed, and the mean values were recorded. The axial length measured by A-scan ultrasonography represents the distance from the front of the cornea apex to the internal limiting membrane. The eye’s refractive status was examined using a streak retinoscope (66 Vision Tech, Suzhou, China) and trial lenses in a dark room. Half an hour before the retinoscopy, one drop of Mydrin-P ophthalmic solution (0.5% tropicamide/0.5% phenylephrine hydrochloride; Santen Pharmaceutical Co., Osaka, Japan) was topically administered every 5 minutes for three times to achieve cycloplegia. Refractive errors of the horizontal and vertical meridians were measured three times, and the means of the measurements were recorded. Intraocular pressure was measured by tonometry (Tono-Pen, Reichert, NY, USA) under topical anesthesia. Three measurements were performed, and the mean values were recorded.

### Tissue Collection and Histologic Preparation

At the end of the study, the guinea pigs and rabbits were anesthetized by inhalation of 5% isoflurane (RWD, Shenzhen, China). For blood sampling, a cardiac puncture was performed, and 2 mL of blood was collected. The blood sample was frozen at −80°C. After sacrificing the animals, the eyes were enucleated. For Western blot and liquid chromatography/mass spectrometry examination, the ocular tissues were harvested separately under the microscope. All samples were immediately stored in liquid nitrogen and transferred to an −80°C freezer. The frozen tissues were analyzed within 1 week.

For histopathologic analysis, the enucleated eyeballs were immediately fixed in 10 mL FAS Eyeball Fixative Solution (Wuhan Servicebio Technology Co. Ltd., Wuhan, China) for 24 hours at room temperature and then embedded in paraffin. Histologic slides (thickness: 8 µm) were prepared and stained with hematoxylin and eosin following a routine protocol. Each eye contained three sections that were obtained along a plane passing through the central cornea, optic nerve head, and the region superior to the optic disc, which was presumed to correspond to the visual streak in guinea pigs.^21^ They were examined under a light microscope (Olympus Co., Tokyo, Japan) by senior ophthalmologists with expertise in ocular pathology (JBJ and WBW). Three histologic sections from each eye were scanned, and the number of cell nuclei in the retinal ganglion cell layer, inner nuclear cell layer, and outer nuclear cell layer was counted.

TUNEL staining (Cell Death Detection kit; Kaiji Biotechnology Co. Ltd., Jiangsu, China) was performed to detect apoptotic cells in the retina. The histologic sections were deparaffinized, and 200 µL Proteinase K (10 µg/mL) was added to completely cover each section for 10 minutes at room temperature, followed by rinsing in PBS three times. The sections were incubated with terminal deoxyribonucleotide transferase enzyme mixture solution (45 µL equilibration buffer, 1.0 µL biotin-11-dUTP, 4.0 µL TdT enzyme) at 37°C for 1 hour and washed with PBS solution three times. Afterward, the sections were labeled with streptavidin-fluorescein (50 µL) for 30 minutes and counterstained with 4,6-diamidino-2-phenylindole (300 nM, 50–100 µL) for 5 minutes. Three sections from each eye were scanned, and the TUNEL-positive cells in the retina were counted. The mean of the counts obtained from three images representing the eyes of different groups was recorded and compared.

### Liquid Chromatography/Mass Spectrometry

High-performance liquid chromatography was used to assess the everolimus concentration in the ocular tissues. Everolimus working standards were prepared by serial dilution into a methanol/water mixture (at 8:2 V/V). The absolute tissue mass was weighed (20 mg), and 100 µL methanol/water mixture was added. After vortex-mixing for 5 minutes, blank tissue samples were mixed with everolimus working standards to generate the standard curves for all kinds of tissue (Supplementary Table S1). The mixtures were vortexed for 5 minutes and centrifuged at 13,200 rpm for 6 minutes. Then, 50 µL of the supernatant was extracted, and we added 150 µL methanol to precipitate the protein. After vortexing for 1 minute, the samples were centrifuged at 13,200 rpm for 6 minutes, and 80 µL of the samples was taken for testing.

Samples were analyzed using an SCIEX Series Triple Quadrupole LC/MS system (SCIEX Co., Framingham, MA, USA) comprising a degasser (SHIMADZU DGU-20A, Shimadzu Corporation, Kyoto, Japan), two binary pumps (SHIMADZU LC-20AD), an autosampler (SHIMADZU SIL-20AC), a column oven (SHIMADZU CTO-20A), a controller (SHIMADZU CBM-20A), and a triple-quadrupole mass spectrometer (SCIEX API 3200MD). Workstation software was used for system control, data acquisition, and data processing. Samples (20 µL) were chromatographed over an Agela Venusil MP C18 column (50 × 4.6 mm, 2.5 µm), at 50°C and with a mobile flow rate of 1.0 mL/min. The runtime was 4 minutes. The gradient mobile phase was (A) organic phase (2 mmol/L ammonium acetate and 1/1000 formic acid) in methanol and (B) aqueous phase (2 mmol/L ammonium acetate and 1/1000 formic acid) in water (all were liquid chromatography/mass spectrometry-grade solvents) as follows: 35% A (0–0.7 minutes), 100% A (0.71– 3.0 minutes), and 35% A (3.01–4 minutes). Everolimus was monitored by positive electrospray ionization on a SCIEX jet stream ion source with ionization source parameters as outlined (Supplementary Table S2). The samples were scanned using multiple-reaction monitoring modes for MRM of everolimus *m/z* [M + NH3]+ (975.6 → 908.6).

### Western Blot Analysis

Frozen RPE–choroid complex tissues were homogenized and lysed in cold lysis buffer (RIPA; Amresco, Solon City, OH, USA) supplemented with protease inhibitors (11697498001; Roche, Basel, Switzerland) and phosphatase inhibitors (04906837001; Roche). The tissue extracts were separated on 8% SDS-PAGE gels and transferred to nitrocellulose membranes according to a standard protocol. The membranes were blocked with 5% skimmed milk in TBST (Tris-HCl, NaCl, and Tween 20) for 2 hours and sequentially incubated with primary antibodies (P70S6K: Immunoway, YM3426, 1:1000 dilution; Immunoway Biotechnology, Plano, TX, USA; p-P70S6K: Abcam, ab2571, 1:1000 dilution; Abcam plc, Cambridge, UK) overnight and with secondary antibodies for 2 hours on the following day. Signals were assessed with an enhanced chemiluminescence (ECL) kit (Millipore, Burlington, MA, USA), and images were taken with the Total Lab Quant V11.5 (Newcastle upon Tyne, UK). The target bands were quantified and analyzed using ImageJ (National Institutes of Health, Bethesda, MD, USA) with β-tubulin as an internal control.

### Statistical Analysis

Statistical analyses were conducted using the Stata 17.0 software program (StataCorp, College Station, TX, USA) and GraphPad Prism 9.4.1 (GraphPad Software, San Diego, CA, USA). Unless stated otherwise, continuous variables are presented as the mean ± standard error. Comparisons of two samples were performed using the two-tailed Student's *t*-test. Paired *t*-tests were used to compare data from the two eyes of individual animals, while unpaired *t*-tests were applied for data from different groups. Comparisons of multiple measurements from the same set of animals at different time points were performed by applying repeated-measures ANOVA and then using a Tukey honestly significant difference post hoc analysis to identify which differences between pairs of means were statistically significant. *P* values <0.05 were considered to indicate statistical significance.

## Results

### Everolimus Eye Drops Formulation Optimization and Its Stability

We first compared several formulations to evaluate their efficiency in delivering everolimus to the RPE–choroid complex. First, we optimized the everolimus eye drops formulation by administering different vehicle formulations that contained 0.001% everolimus once a day for 7 consecutive days. One hour after the final administration, the animals were sacrificed, and the RPE–choroid complex tissues were harvested. Guinea pigs receiving the formulation containing 5% hydroxypropyl β-cyclodextrins, 0.5% methyl cellulose, and 2% polysorbate 80 showed the highest everolimus concentration in the RPE–choroid complex (Supplementary Table S3). Second, this optimized everolimus eye drop formulation demonstrated stability in 4 ± 2°C environments for 12 weeks (98.9% ± 0.3% at week 12 compared to baseline) (Supplementary Fig. S1A). Thus, this vehicle formulation was then used to constitute four different concentrations of everolimus eye drops (0.0001%, 0.001%, 0.01%, and 0.1%).

### Everolimus Eye Drops, LIM, and Fundus Tessellation

**Figure 1.**
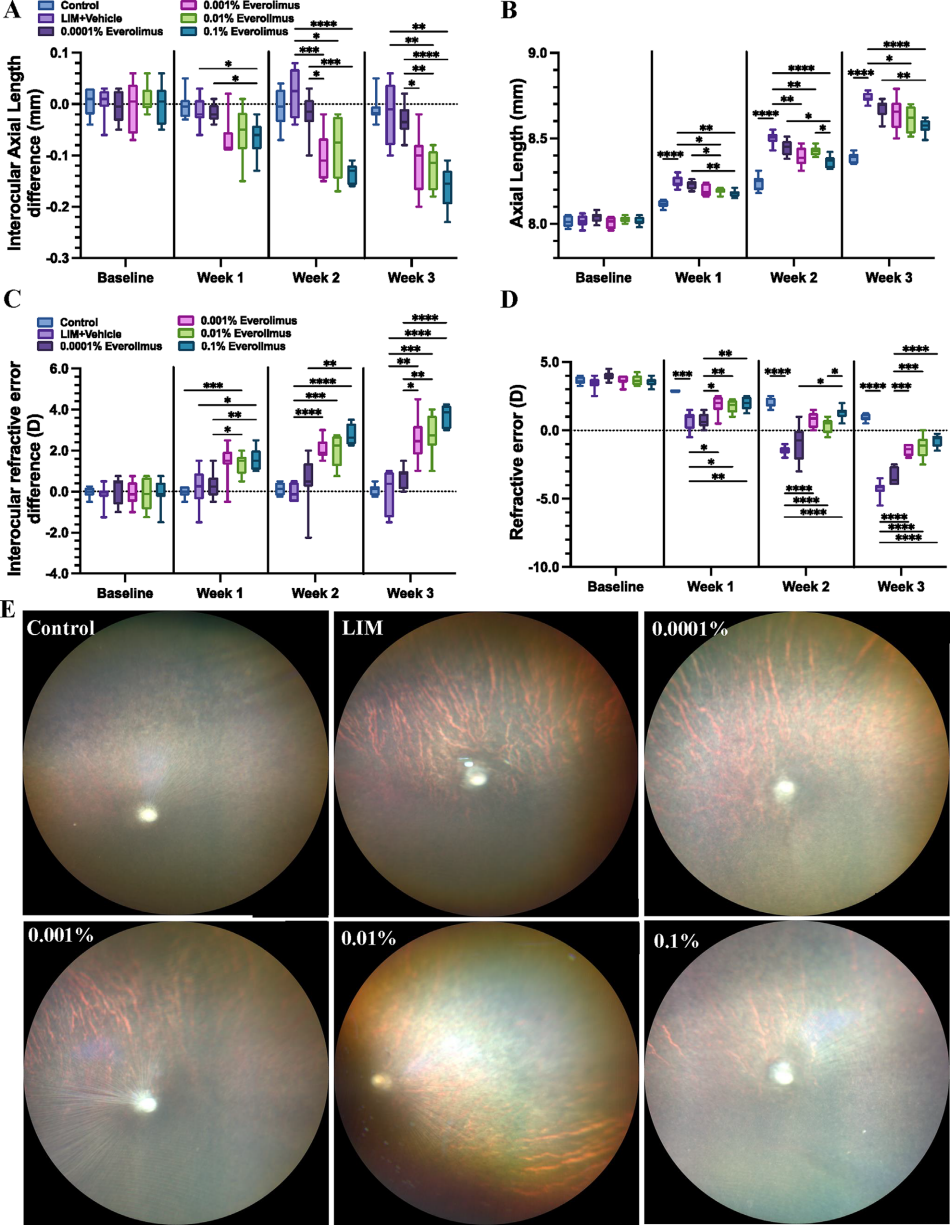
The effect of everolimus eye drops on the development of LIM. (**A**, **B**) Guinea pigs with binocular LIM and monocular everolimus eye drops showed a decreased axial elongation (i.e., a negative interocular axial length difference, *n* = 8). (**C**, **D**) Eyes that received everolimus eye drops showed a smaller myopic refractive shift change under LIM (*n* = 8). (**E**) Fundus images of guinea pigs of negative controls, LIM and vehicle solution without everolimus, and LIM and 0.0001%, 0.001%, 0.01%, and 0.1% everolimus eye drops for 3 consecutive weeks, respectively. All images were taken at the end of the study.

Three weeks of LIM resulted in an increase in axial length by 0.35 ± 0.01 mm (*P* < 0.001) and an increase in myopic refractive error by −5.19 ± 0.23 diopters (D) (*P* < 0.001) (Figs. 1A–D). Compared to the contralateral eyes undergoing LIM alone, daily everolimus eye drop application significantly attenuated axial elongation and myopic refractive shift. At the end of 3 weeks, eyes treated with 0.001%, 0.01%, and 0.1% everolimus eye drops showed smaller axial elongations by 0.10 ± 0.03 mm (*P* = 0.054), 0.11 ± 0.02 mm (*P* = 0.001), and 0.14 ± 0.03 mm (*P* = 0.001), respectively. Correspondingly, eyes receiving 0.001%, 0.01%, and 0.1% everolimus eye drops showed smaller refractive error changes by −2.56 ± 0.53 D (*P* = 0.003), −2.78 ± 0.51 D (*P* < 0.001), and −3.69 ±0.41 D (*P* < 0.001), respectively, than eyes undergoing only LIM. In contrast, the application of 0.0001% everolimus eye drops did attenuate axial elongation (intereye difference in axial elongation: 0.01 ± 0.02 mm; *P* > 0.9) and the degree of myopia (intereye difference in refractive error: −0.72 ± 0.41 D; *P* = 0.54).

Compared to the negative control eyes, well-defined choroidal vessels at the posterior pole were observed in guinea pig eyes with LIM. These well-defined choroidal vessels shared similar characteristics of fundus tessellation in myopic human eyes.^22^ Everolimus eye drops alleviated LIM-related fundus tessellation in LIM eyes (Fig. 1E). Fundus photograph did not show any retinal hemorrhage, exudates, or retinal detachment.

### Ocular Biometric Changes in Guinea Pigs Undergoing LIM and Receiving Everolimus Eye Drops

**Figure 2.**
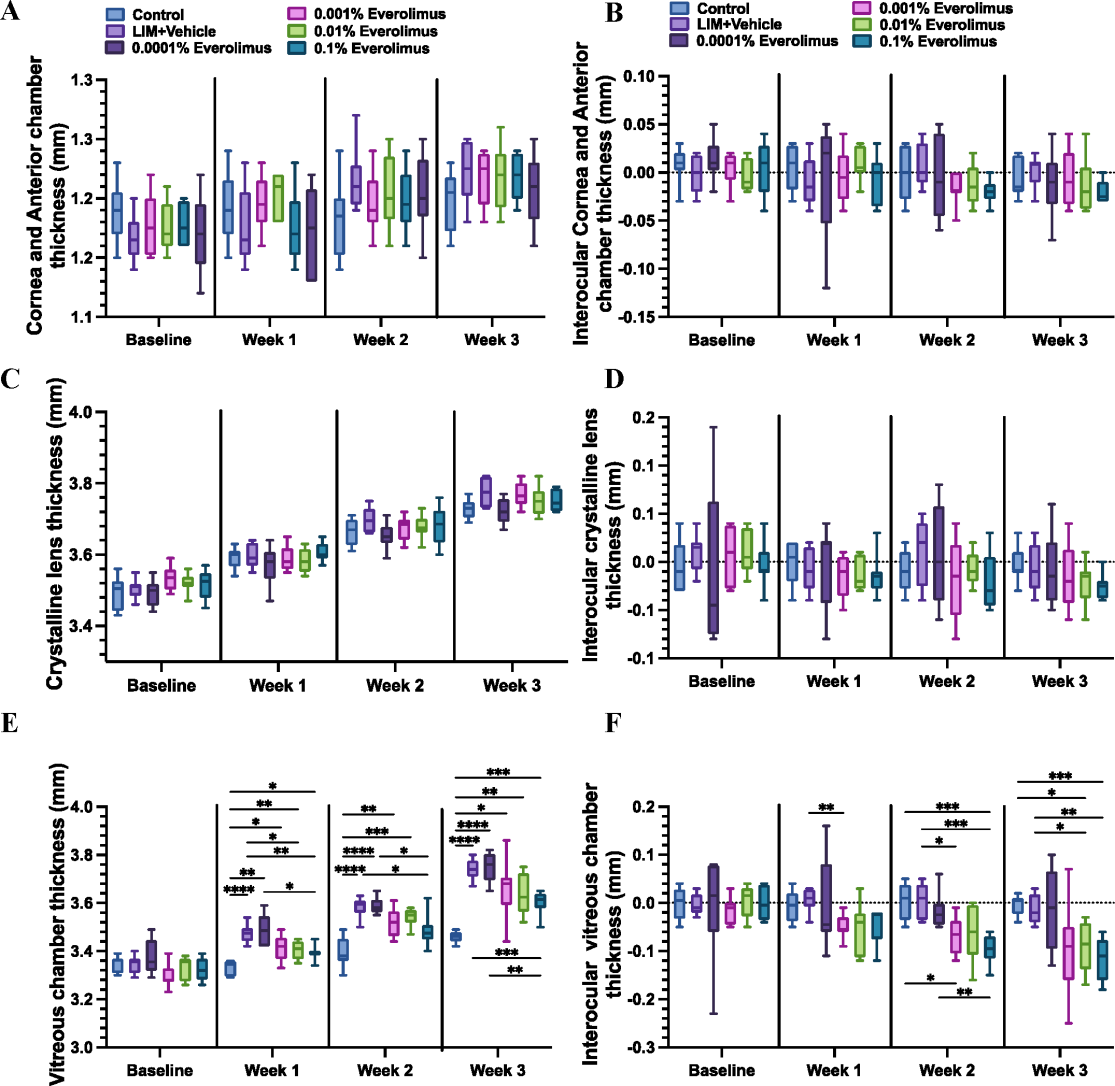
Ocular biometric changes in guinea pigs that underwent lens-induced myopia and received everolimus eye drops. Guinea pigs underwent binocular LIM and daily monocular everolimus eye drops (*n* = 8 for each group). The changes in cornea and anterior chamber depth (**A**, **B**), lens thickness (**C**, **D**), and vitreous chamber thickness (**E**, **F**) were measured.

The changes in axial length were due mainly to changes in the depth of the vitreous cavity. As shown in Figure 2, 3 weeks of unilateral application of 0.0001%, 0.001%, 0.01%, and 0.1% everolimus eye drops reduced the vitreous depth elongation by 0.00 ± 0.03 mm (*P* > 0.9), −0.08 ± 0.04 mm (*P* = 0.31), −0.08 ± 0.02 mm (*P* = 0.04), and −0.10 ± 0.02 mm (*P* = 0.001), respectively, compared to the contralateral eyes undergoing LIM alone. In contrast, unilateral application of everolimus eye drops to LIM guinea pigs did not result in significant interocular differences in cornea and anterior chamber depth combined (0.0001% everolimus eye drops: −0.02 ± 0.01 mm; *P* = 0.88; 0.001% everolimus eye drops: −0.01 ± 0.01 mm; *P* > 0.9; 0.01% everolimus eye drops: −0.01 ± 0.01 mm; *P* > 0.9; 0.1% everolimus eye drops: −0.01 ± 0.01 mm; *P* > 0.9) and interocular lens thickness difference (0.0001%: −0.00 ± 0.02 mm; *P* > 0.9; 0.001%: −0.01 ± 0.01 mm; *P* > 0.9; 0.01%: −0.02 ± 0.01 mm; *P* = 0.78; 0.1%: −0.02 ± 0.01 mm; *P* = 0.45).

### Everolimus Eye Drops and LIM-Related mTORC1 Activation and Choroidal Thinning

**Figure 3.**
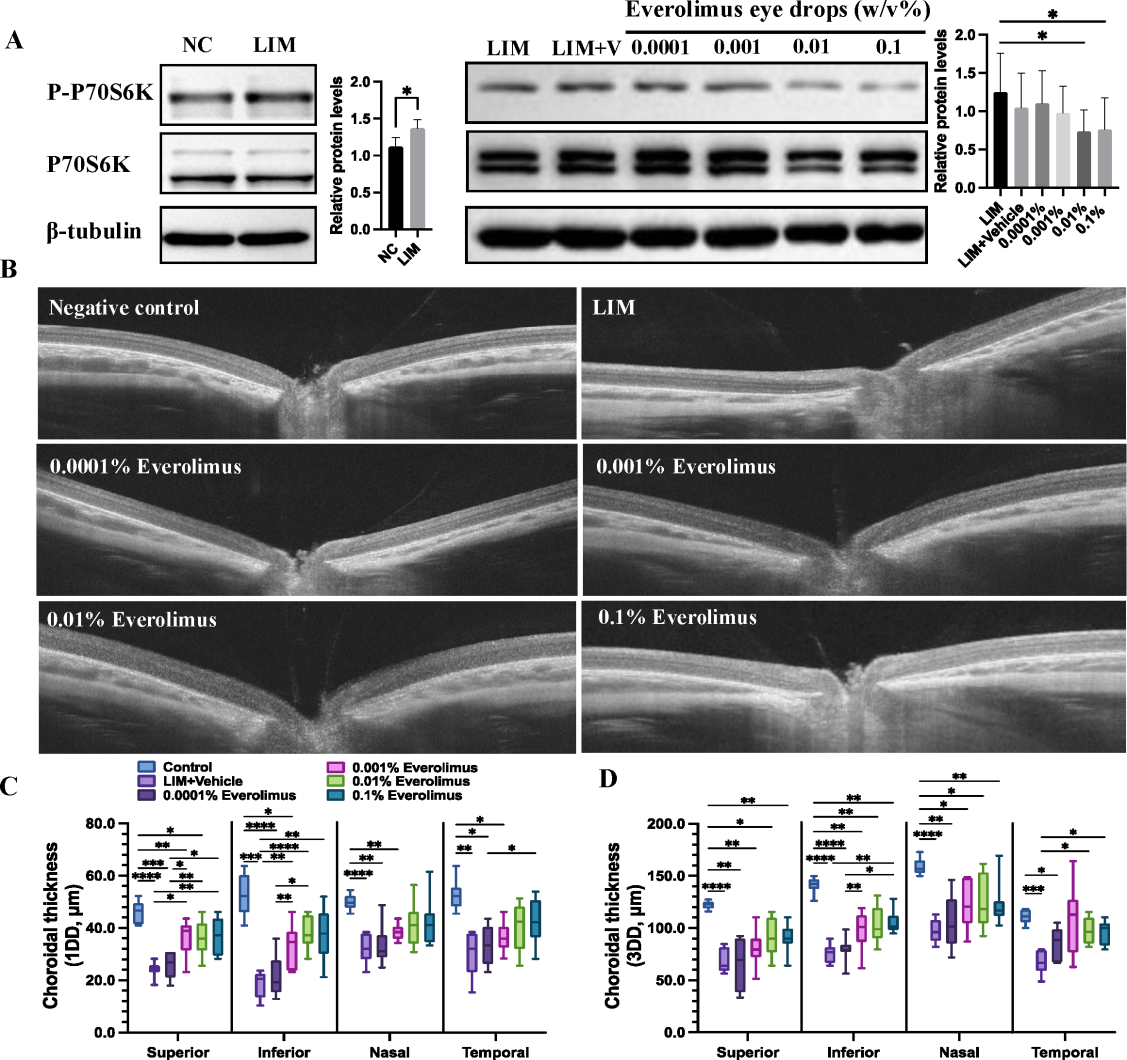
Everolimus eye drops alleviated mTORC1 activation and choroidal thinning in lens-induced myopia. (**A**) RPE–choroid complex tissue was harvested at the end of the study. Compared to normal control eyes, eyes with binocular LIM showed activated mTORC1 (here shown as increased phosphorylation of p70S6 kinase). Topically applied everolimus eye drops were associated with a dose-dependent reduction in mTORC1 activation. (**B**) Representative swept-source OCT images of guinea pigs in negative controls, LIM, and received everolimus eye drops. (**C**, **D**) All images were taken at the end of the study. Choroidal thickness was measured at 1 disc diameter and 3 disc diameters distant from the optic disc (*n* = 8 for each group). DD, disc diameter.

LIM was associated with increased phosphorylation of ribosomal protein S6 kinase β-1 kinase (p70S6K), a key substrate of mTORC1 (Fig. 3A). Application of everolimus eye drops was associated with a dose-dependent reduction in mTORC1 activation (Fig. 3A) and reached statistical significance in the 0.01% everolimus group and 0.1% everolimus group.

Upon OCT, LIM eyes showed a significantly thinner choroid than the eyes of the negative control group (Figs. 3A–C). Application of 0.001%, 0.01%, and 0.1% everolimus eye drops partially attenuated the LIM-induced choroidal thinning in a dose–response manner. Eyes receiving 0.0001% everolimus eye drops did not show significant choroidal thickness differences as compared with eyes undergoing LIM only.

### Safety of Everolimus Eye Drops in Guinea Pigs

**Figure 4.**
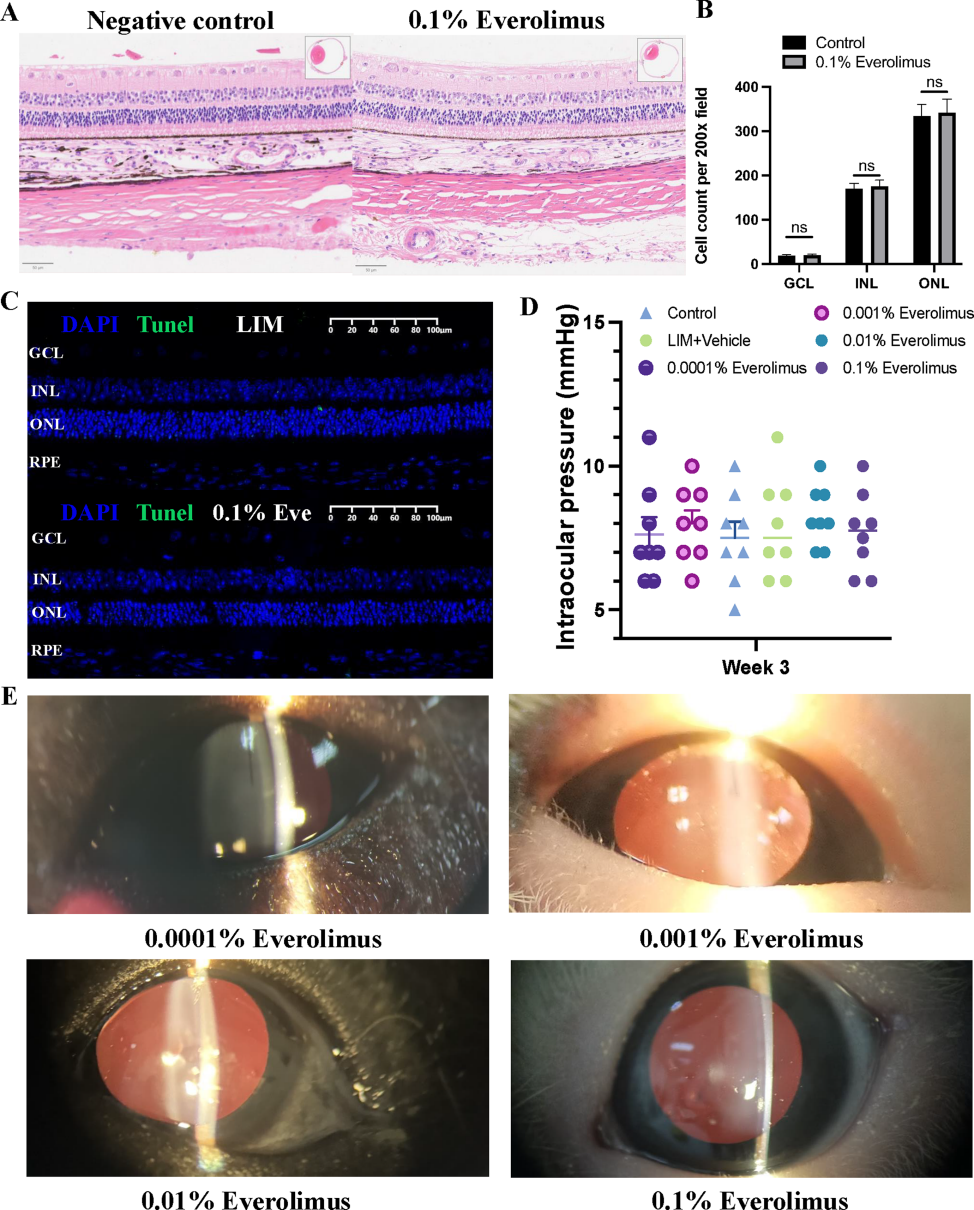
Histopathology, slit-lamp examination, and intraocular pressure. (**A**) Histologic slides, stained by hematoxylin and eosin, of guinea pigs of the negative control group receiving 0.1% everolimus eye drops daily for 3 weeks (*n* = 3). (**B**) Cell count in the retinal ganglion cell layer (GCL), retinal inner nuclear layer (INL), and outer nuclear layer (ONL). (**C**) TUNEL staining of the retina revealed no significant difference in apoptotic cell presence between control eyes and those receiving daily applications of 0.1% everolimus eye drops (*n* = 3). (**D**) Intraocular pressure in the various groups (*n* = 8). (**E**) Slit-lamp examination did not detect corneal erosions, conjunctival hyperemia, keratic precipitate, anterior chamber cells or flare, or cataract formation in eyes that received everolimus eye drops.

After 3 weeks of application of 0.1% everolimus eye drop, the histopathologic examination showed a similar cell density in the retinal ganglion cell layer and in the inner and outer retinal nuclear layers compared to negative control eyes (all *P* > 0.05) (Figs. 4A, 4B). TUNEL staining likewise showed comparable numbers of TUNEL-positive cells between eyes treated with 0.1% everolimus (0.33 ± 0.58 vs. 0.67 ± 0.58 cells per 200× magnification field; *P* > 0.9) and negative control eyes (Fig. 4C). After 3 weeks of application of the eye drops, the intraocular pressure was 7.5 ± 1.6 mm Hg, 7.9 ± 1.7 mm Hg, 7.6 ± 1.7 mm Hg, 8.0 ± 1.3 mm Hg, 8.2 ± 1.0 mm Hg, and 7.7 ± 1.4 mm Hg in the eyes of the negative control group, LIM group, and the eyes receiving everolimus eye drop in concentrations of 0.0001%, 0.001%, 0.01%, and 0.1%, respectively. The differences in intraocular pressure between the groups were not significant (all *P* > 0.2) (Fig. 4D). Slit-lamp examination did not detect lesions or side effects such as corneal erosions, conjunctival hyperemia, keratic precipitate, anterior chamber cells or flare, or cataract formation in eyes receiving everolimus eye drops (Fig. 4E).

### Pharmacokinetics of Everolimus Eye Drops in Guinea Pigs

As the minimum effective concentration of everolimus eye drops was 0.001% for influencing axial length, we additionally explored the single-dose pharmacokinetics of everolimus given in concentrations of 0.001%, 0.01%, and 0.1% in eye drops. The maximal everolimus concentration in the RPE–choroid complex that received one eye drop with everolimus in the concentration of 0.001%, 0.01%, and 0.1% was 5.6 ± 0.5 ng/g (tmax = 1 hour), 13.9 ± 7.8 ng/g (tmax = 1 hour), and 103 ± 40 ng/g (tmax = 1 hour), respectively (Table and Supplementary Fig. S1B). At 24 hours after the application of a single eye drop, the detectable concentration of everolimus suggested an accumulation of everolimus in the RPE–choroid complex. We then examined the repeated‐dose pharmacokinetics of everolimus eye drops by binocularly administering everolimus eye drops once daily for 7 consecutive days. We harvested the RPE–choroid complex and blood samples 1 hour after the final administration. In eyes that received 0.001%, 0.01%, and 0.1% everolimus eye drops, the maximum steady-state concentration in the RPE–choroid complex were 14.1 ± 2.0 ng/g, 21.9 ± 1.2 ng/g, and 141.7 ± 21.5 ng/g, respectively, and the corresponding everolimus concentrations in blood were 0.35 ± 0.08 ng/mL, 4.86 ± 0.24 ng/mL, and 10.20 ± 0.81 ng/mL, respectively (Supplementary Figs. S1C, S1D).

### Tolerability Assessment and Pharmacokinetics of Everolimus Eye Drops in Rabbits

**Figure 5.**
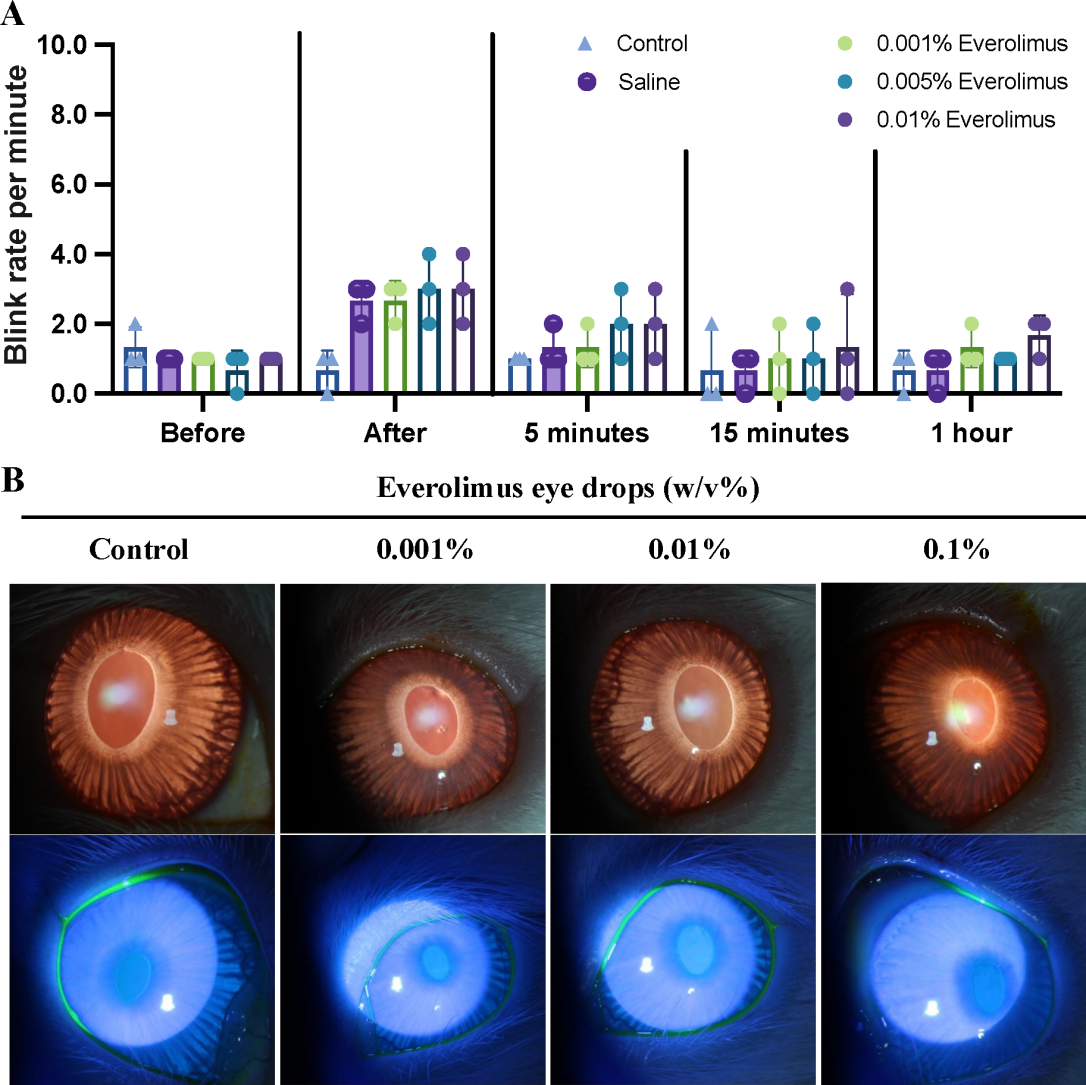
Tolerability of everolimus eye drops in rabbits. (**A**) The average blink rate of rabbits receiving everolimus eye drops (*n* = 3). (**B**) Red reflex images of the crystalline lens and fluorescein staining of the cornea (*n* = 3).

We first calculated the permitted daily exposure (PDE), a presumed dose that is unlikely to cause an adverse effect if an individual is exposed to a substance-specific daily dose for a lifetime.^23^ As calculated in the Supplementary Method, based on longitudinal observation of monkeys and rats, the PDE for everolimus was 6.67 µg/d for general toxication and 12 µg/d for reproductive toxication, equaling 60 µL everolimus eye drops of 0.011% and 0.02% (w/v) per day for a 20-kg child. Thus, three different concentrations of everolimus eye drops (0.001%, 0.005%, and 0.001%) that were below the PDE were repeatedly administered in both eyes of rabbits for 3 consecutive weeks. First, the number of blinks per minute after administering eye drops was recorded. Compared to saline, rabbits that received everolimus eye drops had similar blink rates after eye drop administration (Fig. 5A). After daily administration of everolimus eye drops for 3 consecutive weeks, the fundus red reflex images and fluorescein staining of the cornea surface of all rabbits did not reveal signs of cornea erosion or cataract formation (Fig. 5B).

**Figure 6.**
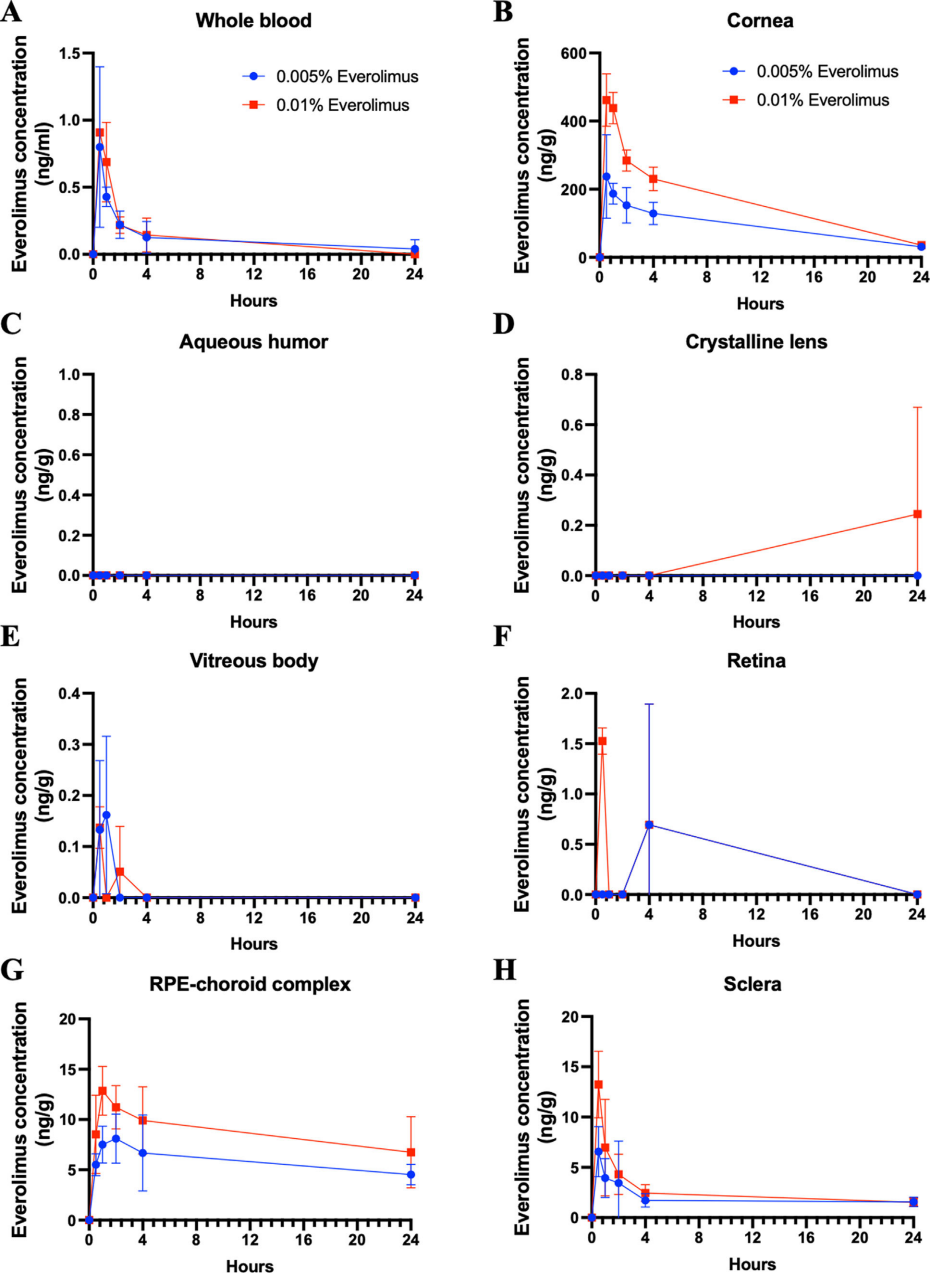
Pharmacokinetics of 0.005% and 0.01% everolimus eye drops in rabbit eyes. Everolimus concentrations were examined at 0.5, 1, 2, 4, and 24 hours after instillation (*n* = 3 per time point). At each time point, eight tissue types were carefully dissected and analyzed separately using HPLC–MS: (**A**) whole blood, (**B**) cornea, (**C**) aqueous humor, (**D**) crystalline lens, (**E**) vitreous humor, (**F**) retina, (**G**) RPE–choroid complex, and (**H**) sclera.

The pharmacokinetics of everolimus eye drops was explored, focusing on the everolimus concentrations of 0.005% and 0.01% in the eye drops, since the prepharmacokinetics data suggested that the everolimus concentration of 0.001% did not show a desirable concentration of everolimus in the RPE–choroid complex (1.68 ± 2.90 ng/g, 1 hour after the eye drop application). Everolimus was mainly distributed in the cornea, followed by the RPE–choroid complex and the sclera (Fig. 6). The trace concentration of everolimus in aqueous humor, crystalline lens, vitreous body, and retina suggested a noncorneal route of everolimus absorption through the conjunctiva and sclera to the RPE–choroid complex. Both 0.005% and 0.01% everolimus eye drops achieved comparable concentrations in the RPE–choroid complex in rabbits and guinea pigs (Table). Repeated‐dose pharmacokinetics showed that for eye drops with an everolimus concentration of 0.005% and 0.01%, the maximum steady state in the RPE–choroid complex was 18.02 ± 6.30 ng/g and 35.98 ± 7.45 ng/g, respectively.

## Discussion

This experimental study developed a hydroxypropyl β-cyclodextrin–based ophthalmic formulation of everolimus eye drops, enabling efficient posterior segment delivery of everolimus, a selective mTORC1 inhibitor, through topical administration. In a guinea pig model of LIM, the optimized formulation of eye drops containing everolimus at concentrations of 0.001% to 0.1% can effectively attenuate myopia progression. Considering that 0.01% represents the upper limit based on the PDE derived from toxicological data, the therapeutic concentration range was therefore defined as 0.001% to 0.01%. Cross-species pharmacokinetics on RPE–choroid complex tissues identified 0.005% to 0.01% everolimus eye drops as the therapeutic window, achieving an equivalent concentration between rabbits and efficacy-proven levels in guinea pigs. Safety analyses showed good tolerance and no evidence of ocular toxicity of the everolimus eye drops in guinea pigs and rabbits.

Emerging evidence positions mTORC1 as a convergent signaling node linking defocus to scleral remodeling and axial elongation in experimental myopia.^12^ It serves as a common secondary signaling, including the epidermal growth factor receptor.^12^ Although mTORC1 has been described as a potential target for myopia control, its therapeutic targeting has been hampered by three translational barriers: (1) low posterior segment bioavailability of conventional formulations, (2) systemic immunosuppression risks, and (3) poor pediatric compliance with intravitreal injections. Our cyclodextrin-engineered everolimus eye drops surmount these limitations that significantly decreased the phosphorylation level of P70S6K. Based on the results of the present study, the administration of everolimus in the form of eye drops appears to fulfill all three goals.

Everolimus is extremely lipophilic, and its log-transformed partition coefficient reaches a value of 5.46, which is markedly higher than the ideal value of 2 to 3 for the permeability of the molecule through the cornea.^24^^,^^25^ The lipophilic characteristics make everolimus tend to remain in the corneal and conjunctival epithelium and to partition only slowly into the posterior eye tissues. To increase the epithelium permeability, several substances are added, including cyclodextrins. Cyclodextrins are used to improve the aqueous solubility of lipophilic drugs with limited solubility in aqueous.^26^ They also reduce the amount of drugs given topically and eventually absorbed into the systemic blood circulation.^26^ Sayed et al.^27^ developed an ophthalmic formulation that delivered the antimycotic and highly lipophilic itraconazole by β-cyclodextrin, achieving significant improvement in corneal permeability. Cytotoxicity studies revealed that α-cyclodextrin in a concentration of more than 4% caused corneal epithelial toxicity, and β-cyclodextrin could lead to a disruption of the corneal epithelium.^28^ In the present study, we tested hydroxypropyl β-cyclodextrins and γ-cyclodextrins, two cyclodextrins that have shown overall safety in ophthalmic formulations so far.^29^ We found that the use of both hydroxypropyl β-cyclodextrins and γ-cyclodextrins could lead to efficient delivery of everolimus to the RPE–choroid complex. The effect of hydroxypropyl β-cyclodextrins on the everolimus delivery was also partially influenced by polysorbate 80 and methylcellulose. Polysorbate 80 acts as a wetting and solubilizing agent, whereas methyl cellulose provides viscosity.^29^

In combination with these substances, everolimus applied in eye drops attenuated the development and progression of experimental myopia in a dose-dependent manner. The pharmacokinetic analysis revealed that everolimus enriched the rabbit RPE–choroid complex. Although the exact mechanism warrants further investigation, observations made in previous studies suggested a role of mTORC1 signaling in the process of axial elongation and determination of choroidal thickness.^12^^,^^30^^,^^31^ Other reports have discussed associations between the mTOR gene polymorphisms and myopia. In a recent study based on 1347 Chinese adolescents with myopia, single-nucleotide polymorphisms (SNPs) of mTOR were found to be closely related to myopia.^32^ Similar findings were also reported by another study.^33^

The regulatory effect of mTORC1 on experimental myopia observed in the present study appears to depend on abnormal visual input, specifically hyperopic defocus. Supporting this, a previous study showed that weekly intravitreal injections of 10 µg everolimus in guinea pigs without LIM did not affect axial length or fundus appearance, suggesting that mTORC1 activation alone is insufficient to drive myopic eye growth in the absence of visual stimulus.^12^ Similarly, environmental factors may modulate the genetic contribution to myopia development. For instance, the genetic effect of amphiregulin (an EGF family member, rs12511037) on refractive error was significantly greater in individuals with higher educational levels compared to those with lower levels, with a mean difference of −0.89 ± 0.14 D. This supports a gene–environment interaction model.^34^ Mechanistically, amphiregulin may promote myopia progression by activating the EGFR–ERK–mTORC1 signaling pathway.^12^ In experimental models, intravitreal injection of amphiregulin increased axial elongation, while intraocular administration of antibodies against amphiregulin and other EGF family members led to a suppression of axial growth.^35^^,^^36^

Based on the findings obtained in the present study, the development of mTORC1 inhibitor eye drops may be of clinical interest also for applications beyond the prevention of myopic progression. Potential indications might include noninfectious uveitis and choroidal neovascularization (CNV). In a clinical phase 3, randomized, double-masked trial, intravitreally applied rapamycin significantly reduced ocular inflammation and improved visual acuity in patients with noninfectious uveitis, with minimal impact on intraocular pressure.^37^ Regarding CNV secondary to age-related macular degeneration, mTORC1 signaling was associated with the CNV development in laser-induced experimental CNV,^38^ which could be inhibited by rapamycin.^39^^,^^40^

The present study has several limitations. First, the short experimental period of 3 weeks might have underestimated the long-term side effects of everolimus eye drops. Long-term administration and observation are needed before any further clinical trials might be considered. Second, we did not assess corneal curvature in the current study. Considering the high concentration of everolimus in the cornea, the biomechanical changes of the cornea might have changed and may be explored in future studies. Third, as with any experimental animal-based study, the results cannot be directly transferred to the clinics.

In conclusion, newly developed ophthalmic formulations efficiently delivered everolimus to the RPE–choroid complex. It significantly attenuated the development and progression of experimental axial myopia and was well tolerated in the context of the study. This proof-of-concept study establishes cyclodextrin-formulated everolimus eye drops as a transformative therapeutic paradigm for myopia control.

## Supplementary Material

Supplement 1
