# Supplementary material for: Topical Application of Everolimus Attenuates Lens-Induced Myopia Through mTORC1 Suppression
Source: Invest Ophthalmol Vis Sci. 2025 Sep 24;66(12):55. doi: 10.1167/iovs.66.12.55 (PMC12476157; doi:10.1167/iovs.66.12.55)
Supplement: Supplement 1 [file iovs-66-12-55_s001.pdf]

Supplementary Materials for

Topical Application of Everolimus Attenuates Lens-induced Myopia Through mTORC1 Suppression

Ruiheng Zhang[1,2,3], Chuyao Yu[1,2,3], Yitong Li[1,2,3], Jiaoyue Dong[1,2,3], Haotian Wu[1,2,3], Yuhang Yang[1,2,3], Xuhan Shi[1,2,3], Wenda Zhou[1,2,3], Hanqing Zhao[1,2,3], Cai Bingyu[4,5,6], , Shanshan Wang[1,2,3], Li Dong[1,2,3], Lei Shao[1,2,3], Wei Li[4,5,6,7], Jost B Jonas[8,9,10,11,12,*], Wenbin Wei[1,2,3,*]

[1] Beijing Tongren Eye Center, Beijing Key Laboratory of Intraocular Tumor Diagnosis and Treatment, Beijing Tongren Hospital, Capital Medical University, Beijing, China

[2] Beijing Ophthalmology & Visual Sciences Key Lab, Beijing Tongren Hospital, Capital Medical University, Beijing, China

[3] Medical Artificial Intelligence Research and Verification Key Laboratory of the Ministry of Industry and Information Technology, Beijing Tongren Hospital, Capital Medical University, Beijing, China

[4] Key Laboratory of Organ Regeneration and Reconstruction, State Key Laboratory of Stem Cell and Reproductive Biology, Institute of Zoology, Chinese Academy of Sciences, Beijing, 100101, China.

[5] Institute for Stem Cell and Regeneration, Chinese Academy of Sciences, Beijing, 100101, China.

[6] University of Chinese Academy of Sciences, Beijing, 101408, China.

[7] Beijing Institute for Stem Cell and Regenerative Medicine, Beijing, 100101, China.

[8] Rothschild Foundation Hospital, Paris, France

[9] Singapore Eye Research Institute, Singapore National Eye Center, Singapore

[10] Privatpraxis Prof Jonas und Dr. Panda-Jonas, Heidelberg, Germany

[11] Beijing Visual Science and Translational Eye Research Institute (BERI), Beijing Tsinghua Changgung Hospital, Tsinghua Medicine, Tsinghua University, Beijing, China

[12] L V Prasad Eye Institute, Hyderabad, Telangana, India

Correspondence to: Wenbin Wei, 1 Dong Jiao Min Lane, Beijing, China 100730. +86-10-58269516; Fax: 86-10-65125617; E-mail: weiwenbintr@163.com.

This PDF file includes:

Materials and Methods
Figures. S1
Tables S1 to S3

Materials and Methods

Permitted daily exposure (PDE) for everolimus.

The PDE is originally used to evaluate the acceptable amounts for residual solvents in the manufacture of drug for the safety of the patient. The everolimus is proved to have general toxication and reproductive toxicology, including immunosuppression, diarrhea, leucocytosis, etc.[1] To minimize toxication of everolimus eye drops, the PDE values were calculated as below to limit the its highest concentration. As the everolimus eye drops were intended for myopia control in childhood, a 20 kilograms of body weight was assumed. The total amount of daily eye drops to the both eye was assumed as 60μL.

**General toxicology, data from monkeys**			**Source**
NOAEL		0.1 mg/kg/day	https://www.accessdata.fda.gov/drugsatfda_docs/nda/2009/022334s000_pharmr.pdf
Body weight		20 kg	
Modifying factors			
	F1	3	F1=3 to account for the extrapolation from monkey to humans
	F2	10	F2 = 10 to account for differences between individual humans
	F3	10	F3 = 10 because the duration of the study was only 52 weeks.
	F4	1	F4 = 1 because no severe toxicity was encountered
	F5	1	F5 = 1 because the no effect level was determined
PDE		6.67	μg/day
Total amount of eye drop		60	μL
Everolimus concentration		0.011%	w/v%

NOAEL, Non Observed Adverse Effect Level

**Reproductive toxicology, data from rat**			**Source**
NOAEL		0.15 mg/kg/day	https://www.accessdata.fda.gov/drugsatfda_docs/nda/2009/022334s000_pharmr.pdf
Body weight		20 kg	
Modifying factors			
	F1	5	F1=5 to account for the extrapolation from rat to humans
	F2	10	F2 = 10 to account for differences between individual humans

F3	1	F3 = 1 for reproductive studies in which the whole period of organogenesis is covered
F4	5	F4 = 5 because teratogenic effect with maternal toxicity
F5	1	F5 = 1 because the no effect level was determined
PDE	12.0	µg/day
Total amount of eye drop	60	µL
Everolimus concentration	0.02%	w/v%

NOAEL, Non Observed Adverse Effect Level

Reference

1 FDA, U. S. https://www.accessdata.fda.gov/drugsatfda_docs/nda/2009/022334s000_pharmr.pdf. (2016).

Figure S1. Stability and pharmacokinetics everolimus eye drops .

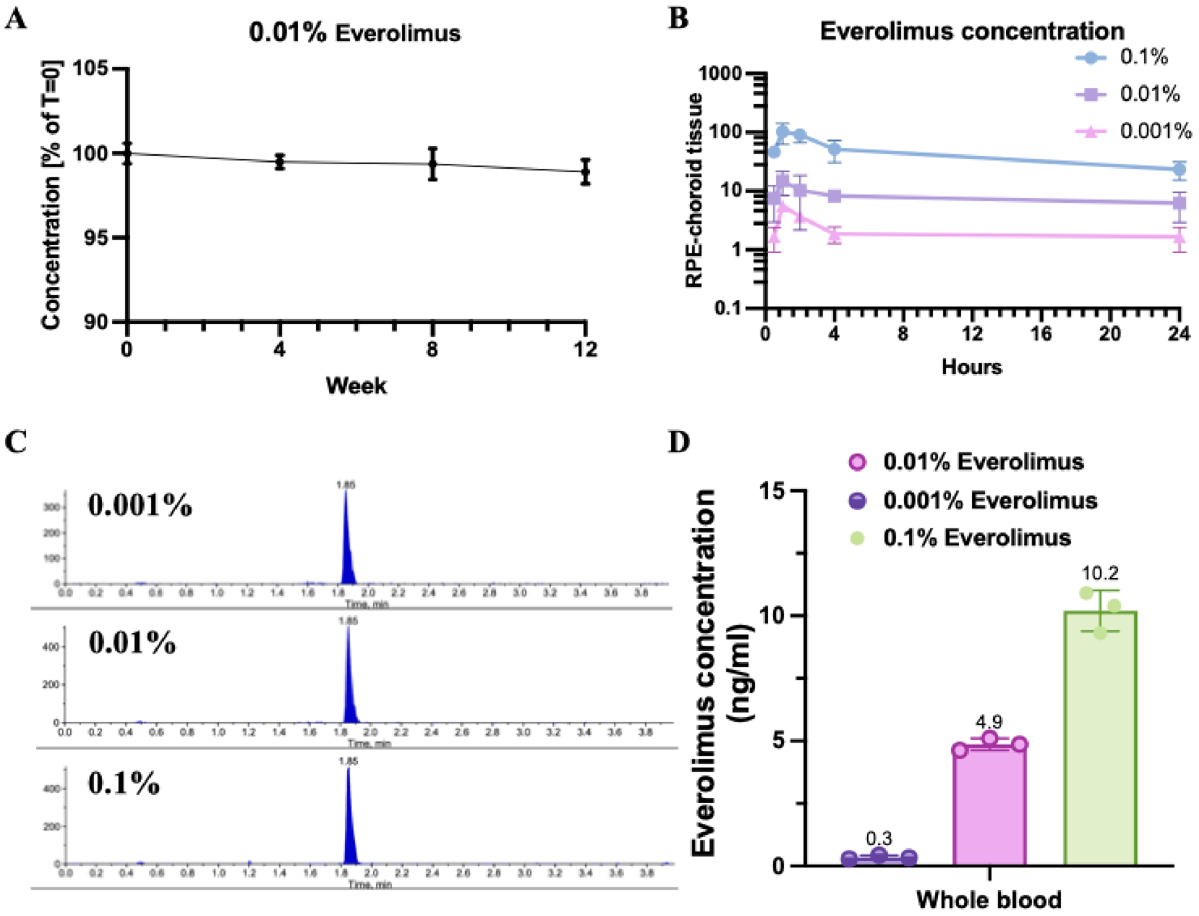

A, 0.01% everolimus eye drops were stored at 4 ± 2 centigrade. The concentration of everolimus were tested at baseline and at 4, 8, and 12 weeks, expressed as percentage to the baseline. B, single-dose pharmacokinetics of 0.1%, 0.01%, 0.001% everolimus in RPE-choroid complex of guinea pigs. C-D, representative chromatogram and concentration of everolimus in the guinea pigs' whole blood.

Supplementary Table 1. LC-MS/MS source parameters for everolimus.

Parameter	everolimus (Analyte)
Capillary voltage (V)	5000
Gas temperature (°C)	550
Air curtain gas (psi)	20
Collision gas	3 Medium
The collision chamber emits pressure	2
Fog gas (psi)	50
Auxiliary gas (psi)	60
Inject voltage (V)	10
Precursor ion (*m*/*z*)	975.6
Product ion (*m*/*z*)	908.6

Supplementary Table 2. Assay parameters of Everolimus in blank and ocular tissues

Parameters	Linearity (ng/mL)	R^2
Guinea pigs		
Whole blood	0.78-75.0	0.9977
RPE-choroid complex	0.78-75.0	0.9992
Rabbits		
Whole blood	0.1-15.0	0.9966
Cornea	1.0-500.0	0.9997
Aqueous humor	0.1-15.0	0.9978
Crystalline lens	0.1-15.0	0.9996
Vitreous body	0.1-75.0	0.9997
Retina	0.1-75.0	0.9999
RPE-choroid complex	0.1-75.0	0.9980
Sclera	0.1-75.0	0.9985

Supplementary Table 3. RPE–choroid complex everolimus concentration after continuously topical administrating different formulation.

Formulation	Hydroxypropyl β-cyclodextrins	γ-cyclodextrins	Methyl cellulose	Polysorbate 80	Everolimus concentration (ng/g)
1	0%	5%	0.25%	2%	12.5 ± 3.6
2	0.50%	0%	0.25%	2%	13.2 ± 1.6
3	2%	0%	0.25%	2%	13.1 ± 1.0
4	5%	0%	0.25%	2%	15.2 ± 2.7
5	10%	0%	0.25%	2%	10 ± 1.1
6	5%	0%	0.00%	2%	11.7 ± 1.1
7	5%	0%	0.50%	2%	9.3 ± 0.2
8	5%	0%	0.25%	0.50%	8.2 ± 1.4
9	5%	0%	0.25%	5%	9.8 ± 1.9
10	5%	0%	0.25%	10%	9.5 ± 0.7

Beside of drugs listed in the table, all formulation contains 0.001% (w/v) everolimus, 0.05mg/ml benzalkonium bromide, and 0.1mg/ml ethylenediaminetetraacetic acid in Sorensen's modified phosphate buffer to adjust PH to 7.4.
